# Large Deformation Finite Element Analyses for 3D X-ray CT Scanned Microscopic Structures of Polyurethane Foams

**DOI:** 10.3390/ma14040949

**Published:** 2021-02-17

**Authors:** Makoto Iizuka, Ryohei Goto, Petros Siegkas, Benjamin Simpson, Neil Mansfield

**Affiliations:** 1 Department of Engineering, School of Science and Technology, Nottingham Trent University, Clifton Lane, Nottingham NG11 8NS, UK; petros.siegkas@ntu.ac.uk (P.S.); ben.simpson@ntu.ac.uk (B.S.); neil.mansfield@ntu.ac.uk (N.M.); 2 Bridgestone Corporation, 1, Kashio-Cho, Totsuka-Ku, Yokohama, Kanagawa 244-8510, Japan; ryohei.goto@bridgestone.com

**Keywords:** polyurethane foam, structure-property relationships, finite element analysis, microscale analysis, X-ray computed tomography

## Abstract

Polyurethane foams have unique properties that make them suitable for a wide range of applications, including cushioning and seat pads. The foam mechanical properties largely depend on both the parent material and foam cell microstructure. Uniaxial loading experiments, X-ray tomography and finite element analysis can be used to investigate the relationship between the macroscopic mechanical properties and microscopic foam structure. Polyurethane foam specimens were scanned using X-ray computed tomography. The scanned geometries were converted to three-dimensional (3D) CAD models using open source, and commercially available CAD software tools. The models were meshed and used to simulate the compression tests using the implicit finite element method. The calculated uniaxial compression tests were in good agreement with experimental results for strains up to 30%. The presented method would be effective in investigating the effect of polymer foam geometrical features in macroscopic mechanical properties, and guide manufacturing methods for specific applications.

## 1. Introduction

**Figure 1:**
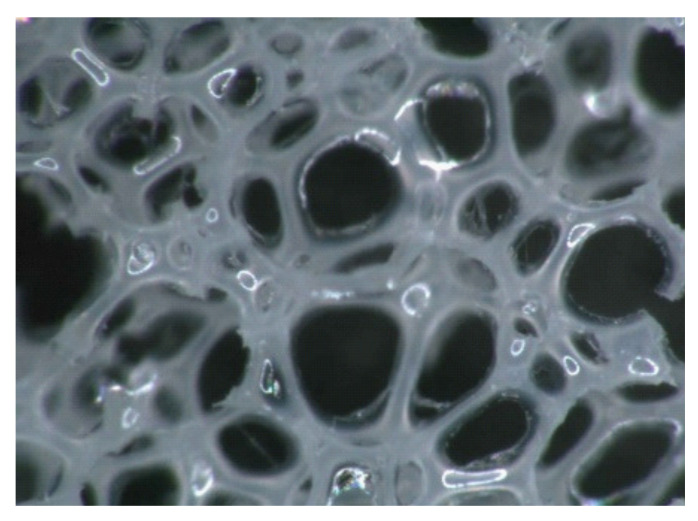
An example of optical microscope images of polyurethane foams.

Polyurethane foams have many unique properties, such as elasticity, softness, and ease of forming. These properties make polyurethane foams attractive to automotive seat designers since they can effectively support the human body and distribute the body pressure. The improvement of the mechanical properties of the foams is an important challenge. Controlling the mechanical properties of foams would be useful in designing seats that are more comfortable and potentially at lower cost. The mechanical properties of polyurethane foams depend largely on their microstructures (Figure 1). The foam structure consists of a cluster of bubbles and struts at the edges of the cells. Figure 1 shows an example of an open-cell foam in which the bubbles are linked together. The macroscopic stress–strain relationship depends on the mechanical properties of the parent material, of which the struts are made, and the geometrical structure of cells and struts [1]. Understanding the relationships between the microscopic geometrical structures and macroscopic mechanical properties is essential in developing foam products with superior mechanical properties.

**Figure 2:**
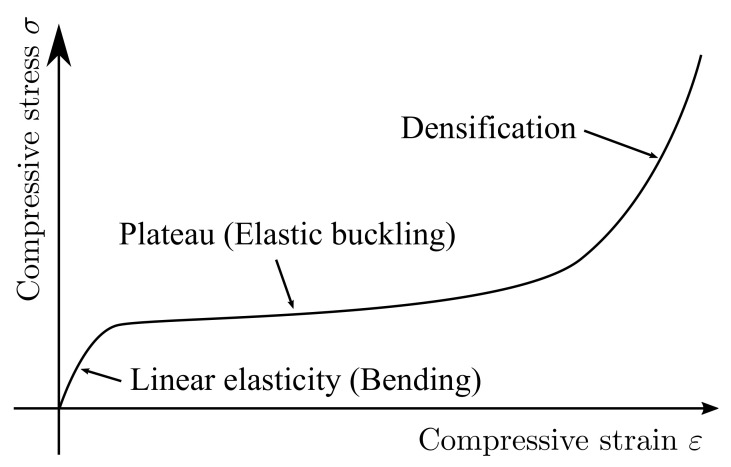
The typical stress–strain relationship of elastomeric foams under the uniaxial compressive stress.

Three main regions can be identified in the stress–strain curve for the compressive deformation of elastomeric foams [1]. Figure 2 shows the typical stress–strain curve under the uniaxial compression of foams. Linear elasticity is shown in the small strain region, followed by a collapse plateau, and then densification appears accompanied by a rapid increase in the stress. Firstly, the struts bend and the macroscopically linear elastic behaviour is shown. Next, some of the struts start buckling and the slope of the curve decreases due to the increase of the macroscopic stress. Finally, the slope of the curve increases again up to the same value as the matrix material, because of the contact between struts. The contribution of microstructures to macroscopic properties depends on these deformation mechanisms.

Cell structure geometries are virtually generated and their deformations are analysed to investigate the effect of microstructures on macroscopic properties [2]. The cells were postulated to have same size and the shape of the Kelvin tetrakaidecahedron. The edges of the polyhedron were assumed to be struts that are represented by Euler–Bernoulli beams and the macroscopic elastic properties were analytically calculated. This approach was also expanded to the large compressive strain range up to 70% [3,4] and creep deformations [5]. Other researchers repeated the calculations of Zhu et al. [2], employing a finite element approach, while still making use of Kelvin’s cell shape and Euler–Bernoulli beams [6,7,8,9,10]. Okumura et al. [11] and Takahashi et al. [12] analysed the mechanical responses in the [001], [011], and [111] directions, as the Kelvin’s cell has anisotropic mechanical properties. Furthermore, closed cell foams have been analysed with shell elements [13]. Modelling the microscopic structures of polyurethane foam materials using the Kelvin’s cell is thought to be a simple and effective way of investigating the deformation behaviour.

The Kelvin cell approach assumes that the microstructure is homogeneous; however, in contrast, cell structures are generally heterogeneous. This is a significant disadvantage of the repeated unit cell modelling approach [14]. To model the inhomogeneous structures of foams, the 2D and 3D Voronoi tessellations were employed and the Voronoi edges were regarded as struts [14,15,16,17]. Moreover, faces in Voronoi polyhedrons were assumed as cell membranes in closed cell foams [18,19]. The elastic properties in the small strain region and the compressive stress–strain curves on the plateau region were calculated by the finite element method while using beam elements. Furthermore, although the cross-sectional area of a strut is often assumed to be constant, the central parts of struts are thinner than other parts. The effect of this necking can be taken into account using solid elements [11,12,20,21,22,23,24,25,26,27] or beam elements with variable cross-sectional properties [23,28,29,30,31,32,33]. In addition, the curvature of struts were modelled [34]. Models that consider the heterogeneity of foams are thought to show better results than Kelvin cell models with straight struts. Dynamic crushing behaviour [35,36] and multiaxial crushing [37] were also analysed.

The use of X-ray computed tomography (CT) is one effective method for obtaining a more adequate model that represents actual foam microstructures. The X-ray CT has been performed to observe the microstructures of various kinds of porous materials, for example, biomaterial scaffolds [38,39], soil materials [40], and polyurethane foams [41]. Therefore, the X-ray CT has also been used to generate the geometries for finite element analyses. For example, finite element models for the microstructure of a trabecular bone was generated based on micro-CT [42]. For artificial foam materials, Jeon et al. [43] analysed closed-cell aluminium foams with finite element models meshed with solid tetrahedron elements. The compressive stress–strain curves of the foam were calculated and compared to the experimental results and the 20.86% volume error was shown up to 5.31% strain. Similarly, linear elastic properties under the small strain regions were obtained from X-ray CT scanned finite element models for ceramic foams [44] and a rigid organic foam [45]. Models that were obtained from the X-ray CT have been effectively used to investigate the mechanical properties of foams under small deformations.

For cushioning products, such as automotive seat pads or bed mattresses, the mechanical properties in the plateau regions are more important than the linear elastic regions. As the slope of the stress–strain curve decreases in the plateau region, elastic foams soften and help to distribute body pressure. Most of the studies employ tetrahedron meshing due to the complexity of the geometry; however, this makes analysing large deformations difficult. To analyse the deformation within the plateau region, hexahedron meshing is required, as it is more suitable for large deformation problems.

This study aims to use X-ray CT scans of foam specimens in order to construct validated finite element (FE) models that can be used to study and manipulate the foam microstructure for achieving desirable stress–strain behaviour in the plateau region. The microstructures of elastic polyurethane foams for automotive seat pads are scanned using X-ray computed tomography and converted to STL files. The STL files are smoothed and converted to solid CAD files with commercial CAD software, so that they can be meshed with a hexahedron dominant solid mesh. The uniaxial compressive deformation of the models are analysed with a finite element method and then compared with the experimental results.

## 2. Materials and Methods

The methodology for analysing the deformation of X-ray CT scanned foam materials and the materials supplied to validate its accuracy are explained here. The specimens were scanned using X-ray CT, converted to CAD models, and then analysed with the implicit finite element method. The tools used for this study are either commercially available CAD or open-source software. Moulded elastic polyurethane foams were investigated using the presented method and physically tested to compare with the result of the analyses.

### 2.1. Materials

The tested materials were supplied by Bridgestone Corporation in Tokyo, Japan. Polyols, isocyanates, water, and low amounts of other materials were mixed and poured into a 400 × 400 × 100 (mm${}^{3}$) sized mould and then expanded and polymerized. After demoulding, the foams were crushed between rollers, so that cell membranes were broken and resulted in open-cell foams. The foams were left at least 24 h before proceeding to any other process of the investigation to let the chemical reactions be completed. The foam materials that were investigated in this study are mainly used for automotive seat pads by moulding in product shaped moulds.

### 2.2. Scanning by the X-ray Computed Tomography

**Figure 3:**
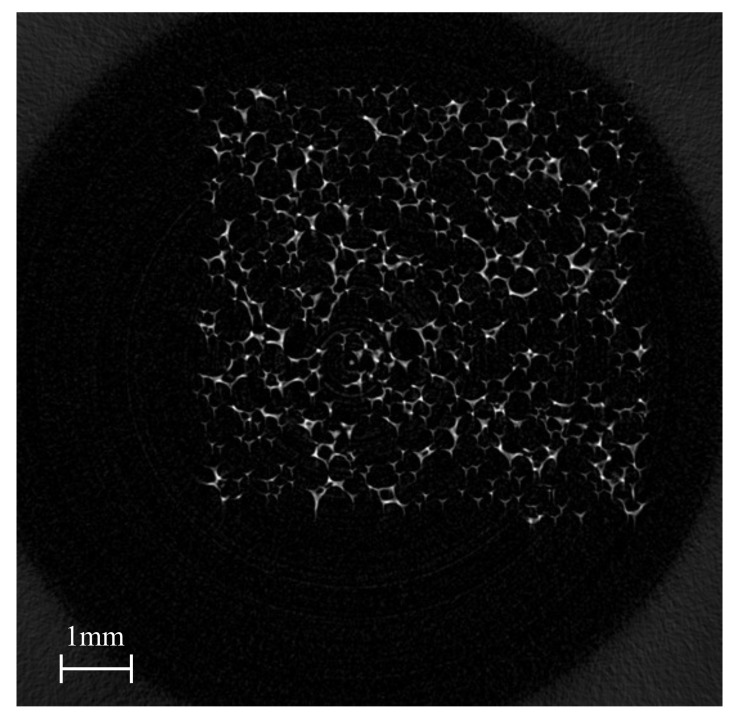
An example of the X-ray computed tomography (CT) scanned images for the polyurethane foams.

Specimens from the centre of larger samples were cut into $5\times 5\times 5\;({\mathrm{mm}}^{3})$ sized cubes. The X-ray tomography equipment that was employed for this study was the ScanXmate RA150S145/2Be, a product of Comscantecno Co., Ltd. in Kanagawa, Japan. Figure 3, shows an example X-ray CT scan image of the foam. The white parts indicate the foam struts and the black parts are the pores. The size of the pixel was 7.5 (μm). The cross section images were taken by rotating the specimens every $0.18^{\circ }$, so that the cell structures could be observed in three dimensions.

### 2.3. Converting the Scanned Images to 3D STL Files

**Figure 4:**
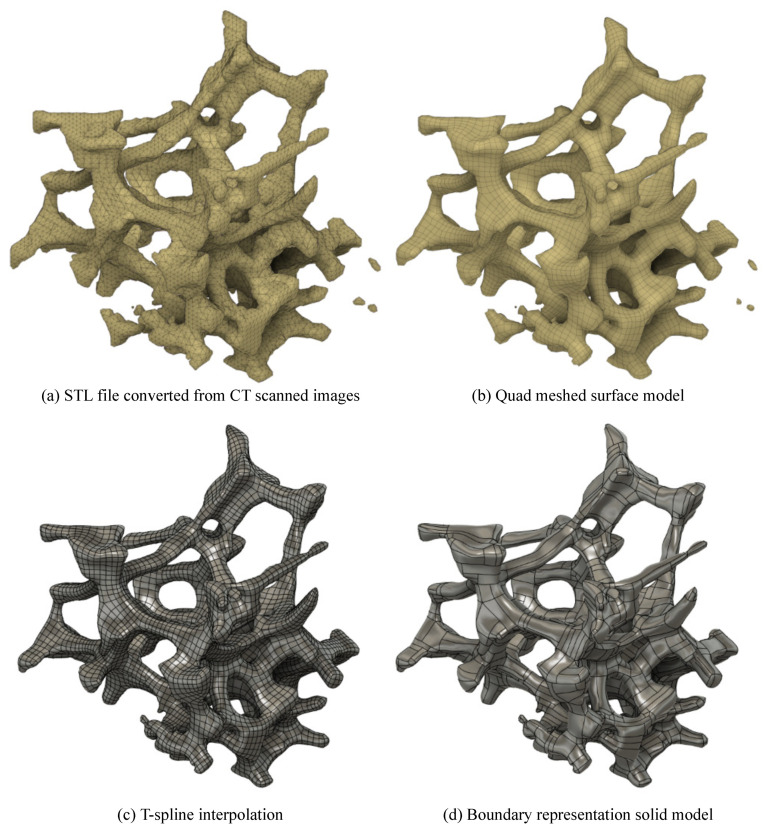
Conversion from the STL files to the boundary representation solid models.

The cross sectional two-dimensional (2D) images were converted to three-dimensional (3D) STL files by Fiji [46], a distribution of Image J2 [47]. Firstly, the scanned images were binarized to black and white images using a threshold of the brightness. The threshold was determined using Otsu’s method [48] and then verified by comparing the relative densities measured with the actual specimen and calculated from the computational models. The borders between the black and white pixels were regarded as the surfaces of the struts. Triangles were then applied to the strut surfaces and the resulting surfaces exported as STL files. Figure 4a shows an example STL file.

### 2.4. Converting to Smoothed Solid CAD Models

The STL formatted files consist of only triangle surfaces and the triangle edges are sharp. When dividing STL files to finite elements directly, the triangle surfaces are divided into further small elements that result in a considerable number of nodes and elements. Therefore, the vertices of the triangle surfaces should be interpolated by mathematically smooth surfaces. This smoothing can be performed using Recap^®^ and Fusion 360^®^ software, both products of Autodesk, Inc. in California, CA, USA. Firstly, the STL files with the triangle meshing were converted to surface models with quad meshing (Figure 4b). The quad meshed surfaces were then interpolated and smoothed by T-spline surfaces (Figure 4c). Finally, boundary representation solid models were generated based on the T-spline surface models (Figure 4d). The resulting solid models were then capable of being analysed in commercial FEA software.

### 2.5. Hexahedron Dominant Meshing

**Figure 5:**
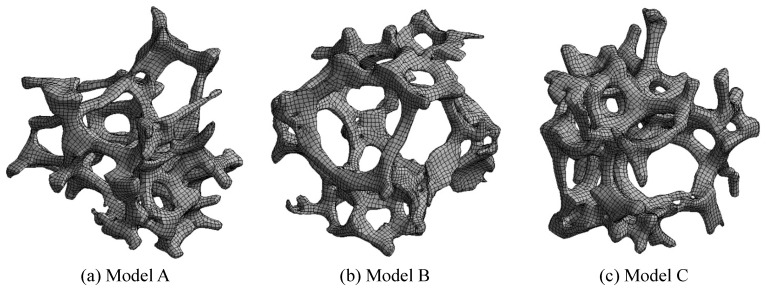
Mesh divisions for the models.

Although the geometries were smoothed by the T-spline interpolation, they were still too complex for hexahedron meshing to be applied. Therefore, mixed hexahedron and tetrahedron meshing was employed. These two kinds of elements were joined by the pyramid mesh elements. The mesh divisions were performed using Ansys^®^ Academic Research Meshing, Release 19.2 [49]. In this study, three representative geometric models were analysed. Figure 5 shows the mesh divisions of these models and Table 1 summarises the numbers of the nodes and the elements in each. Where possible, the models were meshed with hexahedron or pyramid elements.

### 2.6. Finite Element Analyses

The deformation behaviour of three different specimen models was calculated with the commercial FEA software Ansys^®^ Academic Research Mechanical, Release 19.2 [50]. The large deflection was taken into account to analyse the deformations up to the plateau region. As this study focused on the static mechanical properties of polyurethane foams, the static implicit method was employed and the damping or the dynamic characteristics were neglected.

### 2.7. Strut Material Model

A specimen without pores is needed in order to measure the stress–strain relationship of the matrix material. The diameters of the struts are less than 0.1mm and form a complex microstructure. Foam was compressed between plates that were heated to $150{\;}^{\circ }$ C in order to obtain a parent material specimen without pores. The original thickness of the foam was 50mm and the compressed specimen had a thickness of 0.7mm. The measured density of the specimen was $1200\;\mathrm{kg}/m^{3}$.

Tensile testing was performed to obtain the tensile stress–strain relationship. The test equipment was a universal testing machine AGS-X 10 kN with a 500 N load cell, products of SHIMADZU CORPORATION. The specimen was cut into 50 × 5 mm${}^{2}$ rectangular shape specimens and then a tensile test was performed under the strain rate $0.01\;s^{-1}$. The difference between the grippers was regarded as the elongation of the specimen.

**Figure 6:**
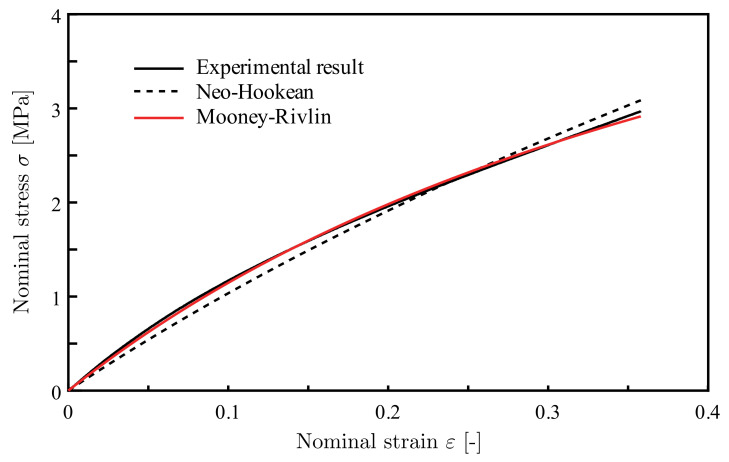
The result of the tensile test for the matrix material and its approximations by hyperelastic models.

Figure 6 shows the measured nominal stress–strain curve. The experimental result is approximated by the neo-Hookean (Equation (1)) and Mooney–Rivlin (Equation (2)) hyper elastic models, respectively.

(1) $$W=C_{10}\left({\bar{I}}_{1}-3\right)+\frac{1}{d}{\left(J-1\right)}^{2}$$

(2) $$W=C_{10}\left({\bar{I}}_{1}-3\right)+C_{01}\left({\bar{I}}_{2}-3\right)+\frac{1}{d}{\left(J-1\right)}^{2}$$

*W* is the strain energy density, ${\bar{I}}_{1}$ and ${\bar{I}}_{2}$ are the first and second deviatoric strain invariants, and *J* is the determinant of the deformation gradient. Table 2 shows the material constants $C_{10}$, $C_{01}$, and *d*. Because the matrix material is thought to be incompressive, *d* was calculated to let the initial Poisson’s ratio ν equal to 0.48. The Mooney–Rivlin model was employed in this study, as it shows better agreement with the experimental result than the neo-Hookean model.

### 2.8. Boundary Conditions

**Figure 7:**
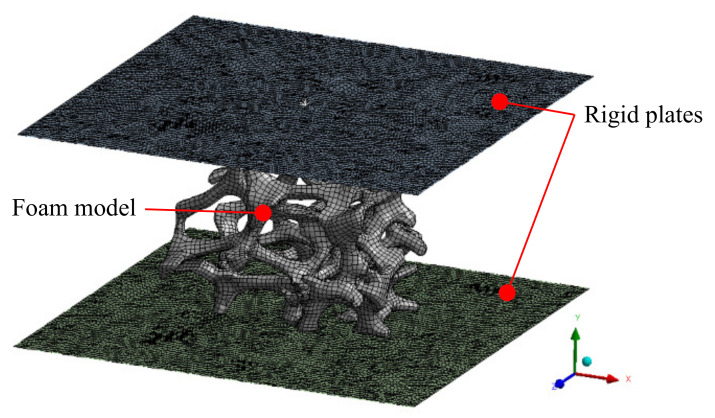
Boundary condition for the uniaxial compression analyses of the foam models.

The foam model specimen was uniaxially compressed between two rigid shell plates, as in Figure 7. The lower plate was fixed preventing any translational or rotational displacements. Translational displacement was applied to the upper plate, whilst all other degrees of freedom were constraint. Frictionless contacts between the foam model and the rigid walls were defined while using the penalty method with a stiffness factor of 0.01. Self-contacts between the struts were not considered, as this study focuses on the buckling behaviour in the transitions to the plateau regions. Finally, remote displacements were used to constraint the specimen lateral boundaries from rigid translational and rotational movement. The average values of the displacements of the nodes on the boundaries that correspond to these directions were fixed. This would allow for deformation, but not rigid body movement.

### 2.9. Experimental Measurement for the Macroscopic Stress Strain Relationships

The uniaxial compression tests for the actual foam specimens were performed to compare with the FEA results. The testing method was similar to ISO3386-1 [51]. The $25\times 25\times 10\;({\mathrm{mm}}^{3})$ sized specimens were cut from the centre parts of the moulded foams. The specimens were set into the same equipment as seen in Section 2.7 with the compression plates. The lower plate was perforated by 6 mm holes arranged in a latticed pattern with 20 mm distances, so that the air in the foam could be ventilated. Firstly, the foams were compressed to achieve 75% nominal strain with the speed 50 mm/s as the pre-compression. Afterwards, the load was taken off with the same speed and the foams were left for 60 s. After that, the foams were compressed again with the same speed and compressive strain to measure the load and the displacement.

## 3. Results

### 3.1. The Deformed Shapes of the Models

**Figure 8:**
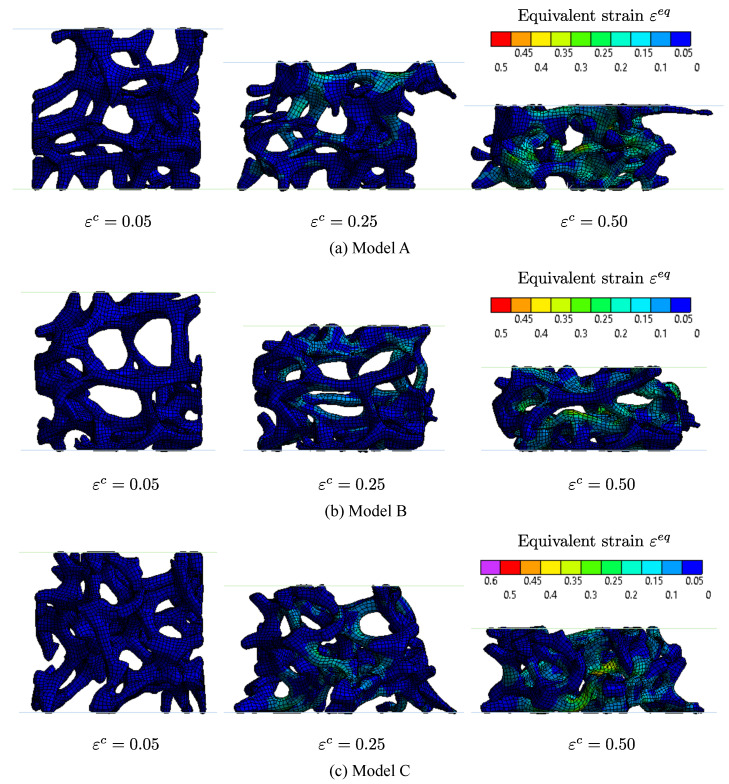
The deformed shapes of the models with the distributions of the equivalent strain $\varepsilon^{eq}$.

Figure 8 shows the deformed shapes of the different specimen models at the macroscopic nominal compressive strains $\varepsilon^{c}=0.05$, 0.25 and 0.50 respectively. The coloured contour represents the Von–Mises equivalent strains $\varepsilon^{eq}$. The struts bend in the linear elastic region ($\varepsilon^{c}=0.05$), as mentioned in Section 1. After that, some struts start to buckle, which indicates a transition to the plateau region ($\varepsilon^{c}=0.25$). Finally, the models gradually become denser and transfer into the densification region ($\varepsilon^{c}=0.50$). Because self-contacts were not applied in the foam models, the struts did not touch, but instead overlapped. The results of the analyses enable the microscopic behaviour of the struts to be carefully observed.

### 3.2. Macroscopic Stress-Strain Relationships

**Figure 9:**
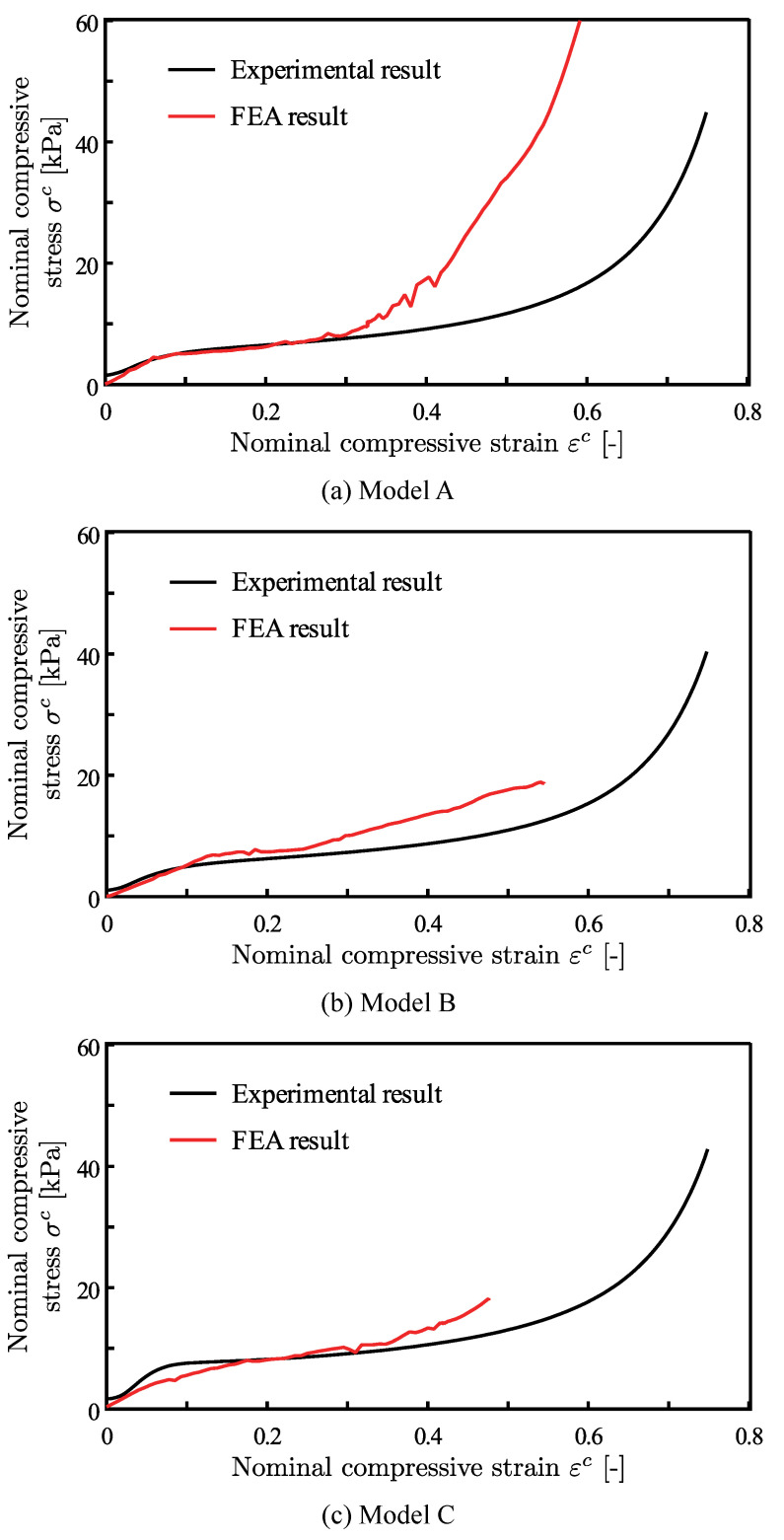
The experimental and FEA results in the relations between the macroscopic compressive stress and strain.

The FEA results were compared with the experiment results to validate the accuracy of the presented analysis method. Figure 9 shows both the experimental and FEA results of the relations between the nominal compressive stress and strain. The slopes of the stress-strain curves for the FEA results start decreasing in the strain region around 0.05 as compared to the smaller strain region. It is thought to mean the transition from the linear elastic regions to the plateau regions.

The models appear to be in good agreement with experiments in the linear elastic and the plateau regions, and up to the strain of 0.30. Differences of the stresses between the experimental and FEA results at the nominal compressive strain of 0.25 were 0.1%, 16.5%, and 6.6% for the models A, B, and C, respectively. In contrast, the FEA results are stiffer than the experimental results in larger strain regions than 0.30. After reaching the strain of 0.30, the slopes of the stress strain curves start increasing again. This behaviour looks similar to the transition to the densification regions; however, self-contacts were not enabled within the model and the stress increase occurs far too early in the strain regions. The presented method should be modified when applied for the densification region.

## 4. Discussions

The compressive response of Polyurethane foam geometries was simulated while using FE methods and then compared with experiments. Foam specimens were scanned using X-ray CT and analysed to obtain geometries for FE simulations. The simulation results were in good agreement with experiments up to 0.3 strain. The finite element model over-predicted stresses, beyond that strain. Three different specimens were scanned and modelled to ensure the repeatability of results.

Elastic buckling appears to be one of the dominant deformation mechanisms. The finite element simulation results seem to have captured the strut deformation behaviour in agreement to relevant literature [1]. Similar deformation mechanisms have been captured with virtually generated cell structures, such as Kelvin’s cells [2,4,8,9,11,12,23,29] or Voronoi polyhedrons [24,27,30,31]. Previous models based on X-ray CT scanned foam structures were mostly limited to small strains (up to 5.31%) [43,44,45].

Hexahedron dominant meshing was used for the large deformation analyses. Struts in foam materials can be long and narrow. Euler–Bernoulli beams have been widely employed for analytical calculations [2,4] and numerical simulations [8,9,24,27,29,30,31]. However whilst beam models might be beneficial in reducing complexity and calculation time, they might also add stiffness to the structure and result in higher stress predictions in comparison to the experimental values. Hexahedron meshes in large deformation problems have been used for simplified geometries [11,12]. The presented smoothing method and hexahedron dominant meshing are recommended for the complex X-ray scanned geometries.

The foam struts at the lateral specimen boundary were unconstraint. Similarly to other studies [31,32,33,36,43], compressive loads were applied in the model by using rigid plates. Contact was defined between the foam specimen and rigid plates. The modelled specimens were smaller than those that were used for experiments. However the boundary conditions seem to have been sufficient in capturing the strut behaviour for strains up to 0.3. The effect of surrounding material at the boundary might have been effectively negligible for up to the strain of interest due to the high porosity of the foam. However, more sophisticated boundary conditions might be required for achieving better accuracy beyond 0.3 strain, or for lower porosity foams. The surrounding cell structures could affect the computed region with bending moments, forces, or contacts between the struts, particularly as the foam densifies. These effects could potentially be taken into account by considering periodic boundary conditions [11,12]. However, this type of boundary condition requires the geometry in the model to be periodic and, therefore, might be more difficult to apply in models of stochastic foam geometries.

**Figure 10:**
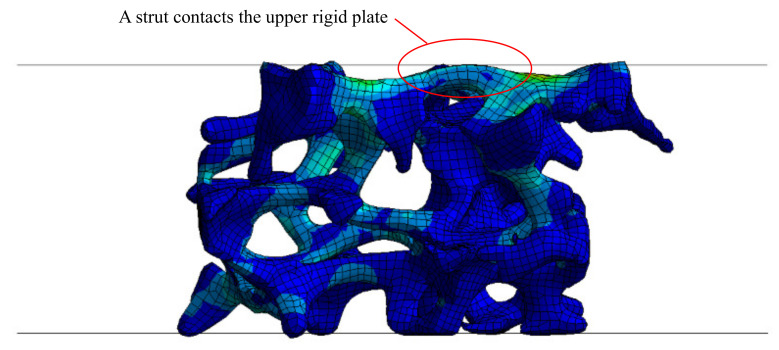
Deformed shape of the model A at the strain of 0.30, when a strut contacts the upper rigid wall.

The finite element model over-predicted stresses, beyond 0.3 strain. Figure 10 shows an example of the deformed modelled specimen at 0.3 strain. As the specimen is compressed, struts that were initially away from the boundary, might then deform and come into contact with the loading plates at the boundary of the specimen. This could cause an increase in the stress response. This could arguably also occur during experiments; however, the model size is considerably smaller than the specimen size in experiments; therefore, the effect of these interactions would be more pronounced in the finite element model simulation. A mitigating approach could be to selectively enable contacts between the loading platens and parts of the foam, i.e., only applying contacts to the nodes on the boundary of the foam rather than the whole specimen. Increasing the model domain size could also improve results. However, a larger model would also increase the computational cost. A damage model was not included in this study. The inclusion of a damage model could potentially improve the accuracy at higher stains.

Analysing the models up to the densification region using implicit FE methods, with the periodic boundary conditions or with larger domains, remains a challenge. Additionally, investigating the effect of strut length and cross-section on the buckling behaviour of struts and the effect of the cell size variation on the linearity of the stress-strain response could inform manufacturing processes for future products. These would be the topics of future work.

## 5. Conclusions

Polyurethane foam specimens, which were intended for automotive seat pads, were scanned using X-ray computed tomography. The scans were converted to 3D CAD models and used to simulate uniaxial compression test using the finite element method. The methodology for the scanning and analyses was described, and the analysis results were compared with the experiments. All three numerical models sufficiently captured the material behaviour in the linear elastic and plateau region of the stress-strain curve. The conclusions for this study are summarised below:

- The investigated foams were scanned by X-ray computed tomography and their structures were captured in 2D cross-section images.
- The observed cross-section images were converted to 3D CAD models using Image J and Autodesk, Inc software products. The smoothed CAD models were analysed with commercial FEA software (Ansys).
- Foam specimens were experimentally tested under uniaxial compression.
- Specimen deformations were analysed by the implicit finite element method with the hexahedron and tetrahedron mixed meshing.
- The mechanical behaviour of foam specimens under compressive loading was sufficiently captured at 0.25 nominal strain and within a reasonable error margin.

The presented method was successfully used to analyse foam structures and it provided a tool in understanding the mechanism of compressive deformations in polyurethane foams. Commercial CAD products and open source software were used for creating a solid mesh for FE analysis from X-ray scans. The chosen approach was perhaps more efficient by comparison to alternative specialised software at a higher cost or in-house development of custom tools. The dependence of the foam macroscopic mechanical behaviour on the microstructural features can now be further investigated to inform manufacturing processes for future polyurethane foam products.

## Figures and Tables

**Table 1:** Numbers of nodes and elements for the models.

	Model A	Model B	Model C
Nodes	27,730	24,937	30,081
Tetrahedron elements	10,873	11,586	12,041
Pyramid elements	16,202	16,795	17,083
Hexahedron elements	13,231	13,746	15,089

**Table 2:** Material constants for the matrix material.

Hyperelasticity Models	$C_{10}\left[\mathrm{MPa}\right]$	$C_{01}\left[\mathrm{MPa}\right]$	$d[{\mathrm{MPa}}^{-1}]$
Neo-Hookean	1.89	-	0.0661
Mooney-Rivlin	0.476	1.78	0.0554

## Data Availability

Data sharing is not applicable to this article.

## Abbreviations

The following abbreviations are used in this manuscript:

CT	Computed tomography
FEA	Finite element analysis
PUF	Polyurethane foam
